# Expression and Significance of CD44, CD47 and c-met in Ovarian Clear Cell Carcinoma

**DOI:** 10.3390/ijms16023391

**Published:** 2015-02-04

**Authors:** Huimin Wang, Mingzi Tan, Song Zhang, Xiao Li, Jian Gao, Danye Zhang, Yingying Hao, Song Gao, Juanjuan Liu, Bei Lin

**Affiliations:** Department of Obstetrics and Gynecology, Shengjing Hospital Affiliated to China Medical University, No. 36 Sanhao Street, Heping District, Shenyang 110004, China; E-Mails: wanghuimin661188@sina.com (H.W.); kitefly1102@126.com (M.T.); ariel890416@126.com (S.Z); lixiaojs@163.com (X.L.); gaoxiaojian@sina.com (J.G.); zhangdanye624@163.com (D.Z.); 18940258190@163.com (Y.H.); song_gao@yeah.net (S.G.); liujj1@sj.hospital.org (J.L.)

**Keywords:** ovarian clear cell carcinoma, CD44, CD47, c-met, immunohistochemistry

## Abstract

Aims: The aim of the present study is to investigate the differential expression of CD44, CD47 and c-met in ovarian clear cell carcinoma (OCCC), the correlation in their expression and their relationship with the biological behavior of OCCC. Methods: We used immunohistochemistry to examine the expression of CD44, CD47 and c-met in OCCC (86 cases) and investigated the effects of the expression and interaction of these molecules on the development of OCCC. Results: CD44, CD47 and c-met expression was significantly high in OCCC. Expression of CD44 and CD47 correlated with patient surgical stage, chemotherapy resistance and prognosis (all *p* < 0.05), and expression of c-met correlated with chemotherapy resistance and prognosis (all *p* < 0.05), but did not correlate with lymph node metastasis (all* p* > 0.05). The surgical stage, CD44, CD47 and c-met expression were independent risk factors for OCCC prognosis (all *p* < 0.05). Patients with low levels of CD44, CD47 and c-met showed better survival than those with high levels (all *p* < 0.05). There was a positive correlation between CD44 (or CD47) and c-met, as well as between CD44 and CD47 (the Spearman correlation coefficient r_s _was 0.783, 0.776 and 0.835, respectively, all *p* < 0.01). Additionally, pairwise correlation analysis of these three markers shows that the high expression of CD44/CD47, CD44/c-met and CD47/c-met were correlated with patient surgical stage, chemotherapy resistance and prognosis (all *p* < 0.05), but did not correlate with lymph node metastasis (all *p* > 0.05). Conclusions: Expression of CD44, CD47 and c-met was upregulated in OCCC and pairwise correlation. CD44, CD47 and c-met may have synergistic effects on the development of OCCC and are prognostic factors for ovarian cancer.

## 1. Introduciton

Ovarian clear cell carcinoma (OCCC), which originates in the Müllerian duct, is a rare pathological type of ovarian epithelial cancer and accounts for 5%–25% of ovarian epithelial cancers. The pathogenetic mechanisms of OCCC remain unclear, although it partly arises from malignant transformation of endometriosis. Compared with other ovarian epithelial cancer subtypes, OCCC presents with characteristic histological and biological changes, such as early staging, higher malignancy, susceptibility to drug resistance, higher risk of relapse and worse prognosis [1,2]. Therefore, understanding the mechanisms of OCCC pathogenesis, pathophysiological course and development may help with early diagnosis, treatment direction and prognosis improvement.

CD44 is a highly heterogeneous transmembrane glycoprotein that is involved in the growth and metastasis of many types of cancer, acting as a cellular adhesion molecule. c-met is a receptor tyrosine kinase. Hepatocyte growth factor (HGF) is the only known ligand of c-met. HGF binds to c-met to form the HGF/c-met system. Studies have confirmed that the HGF/c-met system can activate proliferation, differentiation and adhesion of cancer cells, as well as increase cancer invasion and metastasis. Activation of CD44V6 induces expression of c-met in cancer cells and promotes development, invasion and metastasis of tumors via the CD44V6-c/met/HGF complex, which can activate c-met [3,4]. CD47 is an Ig superfamily member and is also known as integrin-associated protein. CD47 is overexpressed in many malignant tumors, which is closely associated with the malignant biological behavior of tumors, such as immunological evasion. In 2013, Baccelli [5] studied metastasis of circulating tumor cells in the blood of breast cancer patients and reported that metastasis-initiating cells had an EPCAM^+^CD44^+^CD47^+^c-met^+^ phenotype. High expression of CD44, CD47 and c-met increases the risk of metastasis and is negatively correlated with prognosis. The study indicates that CD44, CD47 and c-met are the biomarkers of metastasis-initiating cells of breast cancer. However, only a few studies have investigated the relationship between CD44, CD47 and c-met and ovarian cancer, particularly OCCC. Previous studies have investigated the expression of the three molecules individually in malignant ovarian tumors and have found that CD44 and c-met are highly expressed in OCCC, while CD47 is highly expressed in ovarian cancer tissues. The three molecules are considered to be closely related to tumorigenesis and the progression of cancer [6,7,8,9]. However, studies thus far have not tested the expression of CD44, CD47 and c-met in OCCC simultaneously, as well as the interaction and mechanism of these molecules in the development of OCCC.

This study tests the expression of CD44, CD47 and c-met in OCCC by use of immunohistochemical assays, analyzes the correlation of the expression of the three molecules and investigates the effects of the expression and interaction of these molecules on the development of OCCC. The study provides clues for targeted therapy of OCCC and prognosis improvement.

## 2. Results

### 2.1. Expression of CD44, CD47 and c-met in OCCC 

**Figure 1:**
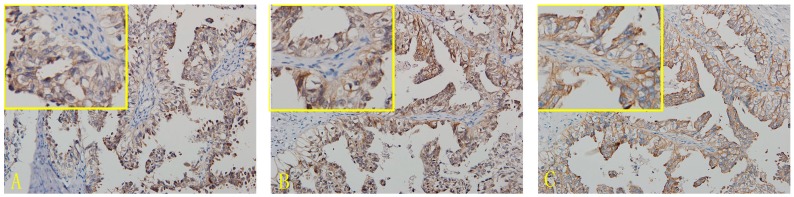
Immunohistochemical micrographs of CD44, CD47 and c-met in ovarian clear cell carcinoma (OCCC) (200×). (**A**) CD44; (**B**) CD47-OCCC; (**C**) c-met-OCCC.

In OCCC tissues, the expression of the CD47 protein was found in the cell membrane and cytoplasm, but mainly in the former. The nuclei were also stained for CD47. The positive expression rate of CD47 in OCCC was 91.9% (79/86). Expression of CD44 was mainly located in the cell membrane, but also found in the cytoplasm, and the positive expression rate of CD44 in OCCC was 90.7% (78/86). c-met protein was mainly expressed in the cell membrane and also found in the cytoplasm. The positive expression rate of c-met was 94.2% (81/86) in OCCC (Figure 1 and Figure S1, Table 1 and Table S1).

**Table 1:** Expression of CD44, CD47 and c-met in ovarian clear cell carcinoma (OCCC).

Groups	Cases	−	+	++	+++	Positive Cases	Positive Rates (%)
CD44	86	8	35	26	17	78	90.7
CD47	86	7	27	37	15	79	91.9
c-met	86	5	21	36	24	81	94.2

### 2.2. Relationship between CD44, CD47 and c-met Levels and the Clinicopathological Features of OCCC

The expression profiles of CD44 and CD47 were similar. The high expression rates of CD44 and CD47 in the early stages of OCCC (Surgical Stage I/II) were 58.3% (35/60) and 50.0% (30/60), respectively, which were significantly lower than in late stages, 69.2% (18/26) and 84.6% (22/26), *p* all < 0.05 (*p* = 0.019,* p* = 0.003). Additionally, the rates of high CD44 and CD47 expression in chemotherapy-resistant patients were 84.2% (16/19) and 84.2% (16/19), respectively, and significantly higher than in chemotherapy-sensitive patients, 61.8% (34/55) and 52.7% (29/55), *p* all < 0.01 (*p* = 0.001,* p* = 0.015). The high expression rates of c-met in patients with Surgical Stage I/II OCCC was 65.0% (39/60), which was lower than in patients with late-stage disease, 80.8% (21/26), but there was no statistical difference between these two groups, *p* > 0.05 (*p* = 0.144). The high expression rates of c-met in chemotherapy-resistant patients was 94.7% (18/19), which was significantly higher than in chemotherapy-sensitive patients 63.6% (35/55), *p* < 0.05 (*p* = 0.010). However, the CD44, CD47 and c-met levels were not associated with lymph-node metastasis (*p* all > 0.05). (Table 2).

**Table 2:** Relationship between CD44, CD47 and c-met levels and clinicopathological features of OCCC.

Characteristics	Cases	CD44			CD47			c-met		
		Low	High	*p*-Value	Low	High	*p*-Value	Low	High	*p*-Value
Surgical stage	86	-	-	0.019	-	-	0.001	-	-	0.144
I/II	60	35	25	-	30	30	-	21	39	-
III/IV	26	8	18	-	4	22	-	5	21	-
Lymph node metastasis *	84	-	-	0.275	-	-	0.288	-	-	1.000
Yes	10	3	7	-	2	8	-	3	7	-
No	74	40	34	-	32	42	-	23	51	-
Chemotherapy	74	-	-	0.001			0.015	-	-	0.010
sensitive	55	34	21	-	26	29	-	20	35	-
resistant	19	3	16	-	3	16	-	1	18	-

* Two cases of ovarian carcinoma patients without lymph node resection.

### 2.3. Risk Factors for OCCC Prognosis

Cox’s regression analysis was performed using surgical stage, lymph-node metastasis, residual lesion size, CD44 expression, CD47 expression and c-met expression as the dependent variables and survival time as the independent variable. The results showed that surgical stage, CD44 expression, CD47 expression and c-met expression were independent risk factors for ovarian cancer prognosis (Table 3).

**Table 3:** Multivariate analysis of the prognosis of patients with OCCC.

Variables	*p*-Value	Hazard Radio (95% CI)
CD44 (low* vs.* high)	0.041	3.249 (1.051–10.048)
CD47 (low* vs.* high)	0.048	4.519 (1.014–20.141)
c-met (low* vs.* high)	0.044	7.981 (1.047–60.240)
Surgical stage (I–II* vs.* III–IV)	0.001	5.612 (2.085–15.103)

### 2.4. Survival Analysis

**Figure 2:**
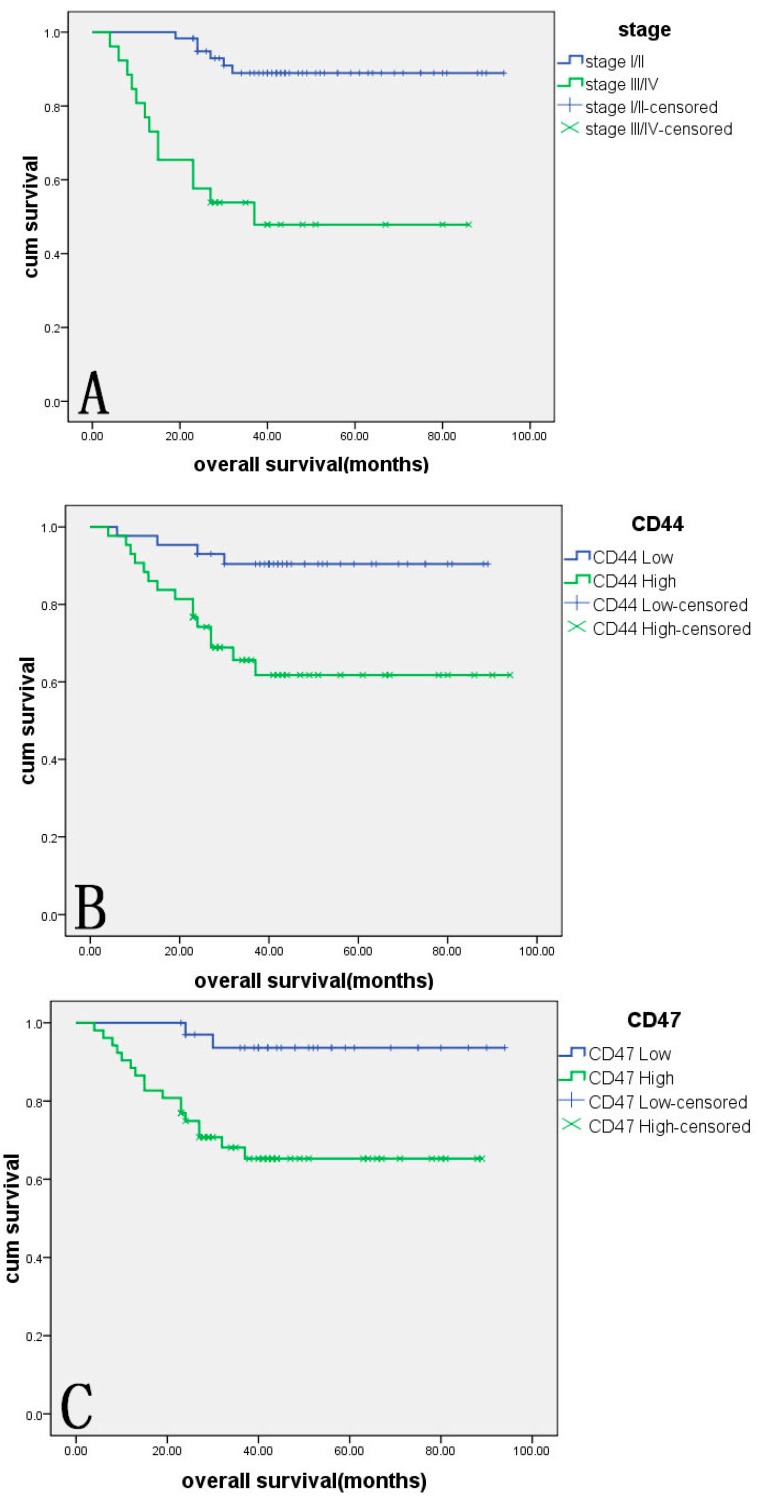

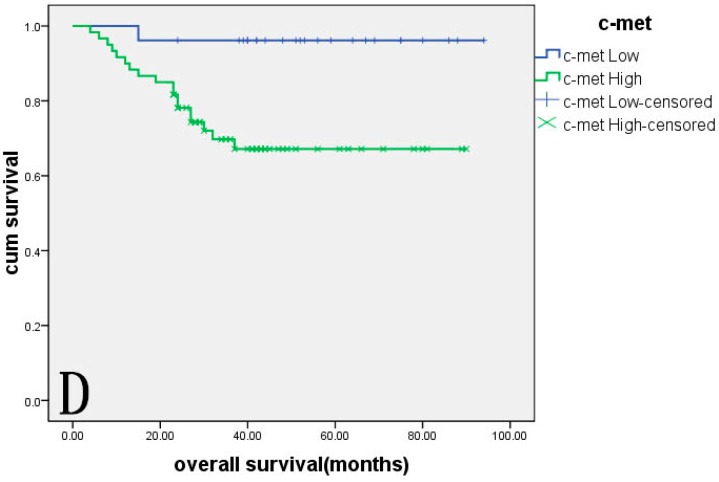
The association between overall survival, CD44, CD47, c-met expression and surgical stage in 86 patients with OCCC. (**A**) Patients with Stage I/II disease showed significantly longer overall survival than those with Stage III/IV disease (*p* = 0.000); (**B**) patients with low CD44 expression showed significantly longer overall survival than those with high CD44 expression (*p* = 0.003); (**C**) patients with low CD47 expression showed significantly longer overall survival than those with high CD47 expression (*p* = 0.003); and (**D**) patients with low c-met expression showed significantly longer overall survival than those with high c-met expression (*p* = 0.007).

As of November 2014, the follow-up time was 23–94 months, and 19 patients died during follow-up. A Kaplan–Meier survival analysis was performed to estimate the survival rates of patients with different intensity of expression of CD44, CD47 and c-met. Results showed that the survival rates of patients with a high expression of CD44, CD47 and c-met were lower than in patients with low expression. The *p*-value of the log rank test was <0.01 in all cases (*p* = 0.003, *p* = 0.003, *p* = 0.007). The survival rate in patients with Stage III/IV was significantly lower than that in patients with Stage I/II disease (*p <* 0.05,* p* = 0.000) (Figure 2).

### 2.5. Correlation between CD44, CD47 and c-met Expression

There was a positive correlation between CD44 (or CD47) and c-met, as well as between CD44 and CD47 in OCCC (the Spearman correlation coefficient *r*_s_ was 0.783, 0.776 and 0.835, respectively, all *p* < 0.01). (Table 4).

**Table 4:** The correlation between CD44, CD47 and c-met expression in OCCC.

CD44	Cases	CD47		c-met		CD47	Cases	c-met	
		Negative	Positive	Negative	Positive			Negative	Positive
**Negative**	8	6	2	5	3	**Negative**	7	5	2
**Positive**	78	1	77	0	78	**Positive**	79	0	79
**Cases**	86	7	79	5	81	**Cases**	86	5	81

Furthermore, pairwise correlation analysis of these three markers shows that the high expression of CD44/CD47, CD44/c-met, CD47/c-met was correlated with patient surgical stage, chemotherapy resistance and prognosis (all *p* < 0.05), but did not correlate with lymph node metastasis (all* p* > 0.05) (Table 5).

**Table 5:** Relationship between CD44/CD47, CD47/c-met and CD47/c-met levels and the clinicopathological features and prognosis of OCCC.

Groups		Surgical Stage			Lymph Node Metastasis *			Chemotherapy			Prognosis		
		I/II	III/IV	*p*-Value	No	Yes	*p*-Value	Sensitive	Resistant	*p*-Value	Live (>3 Years)	Dead	*p*-Value
**CD44/CD47**	CD44-High/	17	17	0.002 *	28	6	0.232 **	14	15	0.002 ***	7	15	0.000 ****
	CD47-High												
	CD44-High/	8	1		8	1		7	1		6	0	
	CD47-Low												
	CD44-Low/	13	5		16	2		15	1		11	2	
	CD47- High												
	CD44-Low/	22	3		24	1		19	2		19	2	
	CD47-Low												
**CD44-c-met**	CD44-High/	23	15	0.024 ^#^	33	5	0.247 ^##^	20	15	0.001 ^###^	10	15	0.000 ^####^
	c-met- High												
	CD44-High /	2	3		3	2		2	1		6	0	
	c-met-Low												
	CD44-Low/	18	6		21	3		16	3		10	3	
	c-met- High												
	CD44-Low/	17	2		19	0		18	0		17	1	
	c-met-Low												
**CD47-c-met**	CD47-High/	20	20	0.011 ^&^	39	7	0.486 ^&&^	24	16	0.002 ^&&&^	14	16	0.000 ^&&&&^
	c-met- High												
	CD47-High/	4	2		5	1		5	0		4	1	
	c-met-Low												
	CD47-Low/	13	2		14	1		11	2		9	2	
	c-met- High												
	CD47-Low/	17	2		18	1		15	1		16	0	
	c-met-Low												
**Cases**		60	26	-	74	10	-	55	19	-	43	19	-

* Two cases of ovarian carcinoma patients without lymph node resection;*, **, ***, **** cases of CD44-high/CD47-high *vs.* CD44-low/CD47-low; ^#^, ^##^, ^###^, ^####^ cases of CD44-high/c-met-high *vs.* CD44-low/c-met-low; ^&^, ^&&^, ^&&&^, ^&&&&^ cases of CD47-high/c-met-high *vs.* CD47-low/c-met-low.

## 3. Discussion

CD44 acts as a cell adhesion molecule and is involved in the regulation of the malignant biological behavior of tumor cells and promotes the growth and metastasis of many types of cancer, including ovarian, breast and colorectal cancer. CD44 realizes its functions through multiple mechanisms, including inhibiting expression of Fas [10], activating the mitogen-activated protein kinase (MAPK) signaling pathway [11], binding to hyaluronic acid and regulating the activity of matrix metalloproteinases (MMPs) [12]. The specific binding of HGF to c-met can induce conformational change of the c-met protein, which triggers the protein tyrosine kinase (PTK) activity of c-met. This is mediated by the intracellular kinase domain of c-met, which triggers the trans-phosphorylation of c-met. PTK can further catalyze tyrosine phosphorylation of a variety of substrate proteins. The signal is amplified by a cascade reaction and transduced into the nucleus to regulate gene transcription. The c-met signal transduction system plays a key role in regulating the proliferation, differentiation, motility and migration of tumor cells. Abnormality of the c-met pathway can increase the aggressiveness of tumor cells and induce angiogenesis. Studies regarding the relationship between CD44 and c-met have shown that stimulation of CD44V6 induces expression and activation of c-met, thus promoting activation of MMP-9 by upregulating expression of urokinase-type plasminogen activator (uPA) through the Ras signaling pathway [13]. uPA can degrade extracellular matrix and facilitate invasion and metastasis of cancer cells. HGF can amplify lymphocyte function antigen (LFA)-mediated adhesion of cells prestimulated by CD44V6 and further trigger and amplify strong, integrin-mediated adhesion. This results in the adhesion of cancer cells to vessel endothelial cells and subsequent trans-vessel wall migration [3]. Rousseau [4] found that the extracellular domain of CD44V6 is required for autophosphorylation of c-met. The formation of the CD44V6/c-met/HGF complex can promote the phosphorylation of receptor tyrosine kinase c-met. Phosphorylated PTK interacts with Fas and inhibits binding of Fas to its ligand, which promotes cell proliferation and inhibits apoptosis. Activated c-met can activate the phosphatidylinositol 3-kinase and MAPK signaling pathway and upregulate expression of pro-angiogenesis factors interleukin-8 and vascular endothelial growth factor to induce angiogenesis and boost the development and metastasis of cancer [14]. CD44V6 can also mediate binding of HGF to the methionine receptor, promoted by heparan sulfate binding growth factor and stimulate metastasis. Damm [15] suggested that HGF stimulates the secretion of CD44 by melanoma cells by activating the nuclear factor-κB signaling pathway. Blockade of CD44 with monoclonal antibodies can inhibit activation of c-met and the potential migration of cells induced by HGF. This confirms that cell motility stimulated by HGF is based on overexpression of CD44. CD44 plays a significant role in the activation of the HGF/c-met signaling system. Chuan [16] discovered that ezrin is a downstream molecule of c-met. c-met can influence the motility and invasion of cells that are related to metastasis through regulating the expression and distribution of the cytoskeleton and cellular adhesion molecules. Ezrin can directly bind to CD44V6, thus making CD44V6 bind to the cytoskeleton indirectly, to regulate cell migration and morphology and strengthen metastatic potential [17]. CD44 and c-met are supposed to promote the development of tumor malignant behavior synergistically. Our study showed that CD44 and c-met were both highly expressed in 86 cases of OCCC; the positive expression rates were 90.7% (78/86) and 94.2% (81/86), respectively, and were higher than the positive rates (72.7%, 16/22; 86.4%, 19/22) in 22 cases of other ovarian cancer subtypes, consistent with the literature [18,19]. However, the cases of other ovarian cancer subtypes, except OCCC, were less (only 22 cases) in our experiment, and continuing research will be important to confirm the different expression of these three markers in different ovarian cancer subtypes. Expression of CD44 and c-met was associated with the clinical stage of OCCC, chemotherapy resistance and prognosis. In OCCC, there was a positive correlation between the expression of CD44 and c-met. Thus, we assume that the interaction between CD44 and c-met plays an important role in the development and progression of OCCC.

CD47 performs its regulatory functions by interacting with its ligands. CD47 can regulate chemotaxis, adhesion, cytophagy and migration of neutrophils and activate the host inflammatory response against infection by binding to integrin. The binding of CD47 to integrin can also induce the activation and aggregation of platelets and promote the proliferation and migration of smooth muscle cells. Binding of CD47 to thrombospondin (TSP)-1 can regulate cell proliferation, apoptosis, adhesion and migration and participates in the process of angiogenesis and the inflammatory response. Binding to signal regulatory protein α (SIRPα) can transduce inhibitory signals to decrease the phagocytic activity of macrophages and boost the immunological evasion of tumor cells, as well as tumor development [20]. Kim [21] found that CD47 may play an inhibitory role in NK cell-mediated cytotoxicity against cancer cells, implying a possible mechanism of immune escape in human cancer. CD47 is expressed on the surface of all human solid tumor cells, and binding to SIRPα blockade of CD47 signaling using targeted monoclonal antibodies enabled macrophage phagocytosis of tumor cells that were otherwise protected* in vitro* [22]. Our results show that CD47 was mainly found in cancer cells in OCCC, and combined with those studies mentioned above, we speculate that cancer cells of OCCC overexpress CD47, then bond with its ligand, SIRPα, and reduce the phagocytosis function of the macrophage, thereby promoting OCCC occurrence. Overexpression of CD47 has been found in tumor stem cells, including acute myeloid leukemia and non-Hodgkin’s lymphoma. High expression of CD47 is associated with poor prognosis [23]. CD44^+^ cells have been found in breast [24] and gastric cancer [25] in spite of a relatively small fraction in tumor cell populations. CD44 is considered as a surface biomarker of tumor stem cells for CD44^+^ cells with self-differentiation and proliferation, renewal and antiapoptotic potential. Tumor-initiating cells (TICs; lineage CD44^+^, CK5^+^, CK20^−^) in human bladder cancer have been isolated and a cell line established. It has been found that TICs have strong proliferative ability and can form xenografts* in vivo*. Expression of CD47 in CD44^+^ subpopulations of TICs is significantly higher than in CD44^−^ subpopulations. Histological tests for bladder cancer primary transplant nodules in nude mice showed that CD44 and CD47 were highly expressed in the outer rim of cells in tumor nodules, but most internal cells in the nodules are CD44-low and CD47-low. CD44 and CD47 are both supposed to be biomarkers of bladder cancer TICs [26,27]. Our results indicated that expression of CD47 was comparable to expression of CD44 in different ovarian cancer subtypes and displayed a positive correlation. Expression of CD44 and CD47 was significantly higher in OCCC and associated with clinical stages, chemotherapy resistance and prognosis. We suppose that CD44 and CD47 are both biomarkers of OCCC stem cells. They synergistically promote proliferation and differentiation of stem cells and tumor development. 

Only a few studies have investigated the relationship between CD47 and c-met. Scarpino* et al.* [28] found, in thyroid papillary carcinoma, that expression of TSP-1, a CD47 ligand, was regulated by c-met/HGF. CD47 prevents the adhesion of cultured MDCK cells, while HGF stimulates their adhesion. CD47 and c-met synergistically accelerate cell migration by remodeling the cytoskeleton [29]. In our study, CD47 and c-met had similar expression features. They may interact with each other in the development of OCCC.

In 2013, Baccelli [5] studied the metastasis of circulating tumor cells in the blood of breast cancer patients and reported that metastasis-initiating cells had an EPCAM^+^CD44^+^CD47^+^c-met^+^ phenotype. High expression of CD44, CD47 and c-met increases the risk of metastasis and is negatively correlated with prognosis. The study indicates that CD44, CD47 and c-met are the biomarkers of metastasis-initiating cells of breast cancer. Our study indicated that expression of CD44, CD47 and c-met was upregulated in OCCC and displayed similar expression features. Expression levels of the three molecules were associated with clinical stage, chemotherapy resistance and prognosis of OCCC. Higher levels of CD44, CD47 and c-met implied a later stage, higher risk of developing chemotherapy resistance and poorer prognosis. Patients with negative expression of CD44, CD47 and c-met had better prognosis. One Stage IIIA patient in whom CD44, CD47 and c-met showed negative expression had no signs of recurrence after three years of follow-up after systemic therapy. The results suggest that CD44, CD47 and c-met participate in the development of the malignant biological behavior of OCCC and promote tumor progression and poor prognosis. Further analysis showed a positive correlation between CD44 (or CD47) and c-met, as well as between CD44 and CD47. Additionally, pairwise correlation analysis of these three markers shows that the high expression of CD44/CD47, CD44/c-met, CD47/c-met was correlated with patient surgical stage, chemotherapy resistance and prognosis (all *p* < 0.05). This implies that CD44, CD47 and c-met may have synergistic effects on the development of OCCC and probably are biomarkers of tumor stem cells of OCCC. Further studies are needed to elucidate the effects of CD44, CD47 and c-met on the malignant biological behavior of OCCC cells, including proliferation, apoptosis, adhesion, invasion and metastasis. Research is also needed to determine by which pathways these molecules regulate the behavior of cancer cells, as well as the interaction among these molecules, to provide a theoretical basis for exploring the diagnosis and improving the prognosis of OCCC.

To date, drugs targeting CD47, CD44 and c-met have been developed to treat breast, lung, colon and gastric cancer and leukemia. Clinical trial results show that they have certain antitumor activities and can improve prognosis. Results from several large clinical trials indicate that monoclonal antibodies have synergic effects when used in combination with chemotherapy drugs and can improve efficacy and survival. Combination regimens will be a trend in the future. OCCC is a subtype of ovarian cancer that is notorious for its high malignancy and inclination towards chemotherapy resistance and poor prognosis. Our study suggests that CD47, CD44 and c-met may have synergistic effects on the development of OCCC. Drugs targeting CD47, CD44 or c-met can be used in combination with standard chemotherapy. This is significant for personalized medicine and prognosis improvement of OCCC.

## 4. Experimental Section

### 4.1. Patients and Tissue Samples

Eighty one OCCC tissue samples were obtained from operations performed from 2006 to 2012 in the Department of Gynecology and Obstetrics of Shengjing Hospital Affiliated with China. All tissue sections were examined by specialists to obtain a final diagnosis. All cases were primary, and the information was complete. All of the patients had received gastroscopy or colposcopy to exclude other primary tumors. Patients were not subjected to chemotherapy prior to surgery. The clinical and pathological information about the patients was collected from their clinical records and included their age, surgical stage, lymph-node metastasis and tumor subtype. The age range of the OCCC group was 29–73 years, with a median of 50.3 years. The classification of cancer stage was according to the International Federation of Gynecology and Obstetrics (FIGO, 2009). The clinical and pathological information about the patients was collected from their clinical records and included their age, surgical stage, lymph-node metastasis and residual tumor size. In total, 8 patients were censored; 4 patients did not take any chemotherapy after surgery; and 74 patients, who received follow-up, were treated with systemic chemotherapeutic regimens based on paclitaxel plus platinum according to National Comprehensive Cancer Network (NCCN) guidelines. The patients were divided, according to NCCN guidelines, into a resistant or sensitive group. The resistant group, initially treated with chemotherapy based on carboplatin and paclitaxel, achieved clinical remission, but had cancer recurrence during the late stage of chemotherapy or within 12 months post-chemotherapy. The sensitive group included patients with clinical remission over 12 months. By this standard, 55 of 74 patients were chemotherapy sensitive, and 19 were resistant.

### 4.2. Methods

#### 4.2.1. Immunohistochemistry

Histological sections of 5 mm were taken from each group of ovarian tissues. Each tissue had two serial sections. Expression patterns of CD44, CD47 and c-met in OCCC tissues were analyzed via immunohistochemical streptavidin-peroxidase staining. Positive and negative immunohistochemistry controls were routinely used. Breast cancer tissue was used as the positive control for CD44 and CD47, and endometrial cancer tissue was used as the positive control for c-met. The negative control was incubated with phosphate-buffered saline instead of primary antibody. The working concentrations of primary antibodies against CD44, CD47 and c-met used were 1:100 (rabbit polyclonal antibody; Santa Cruz Biotechnology, Dallas, TX, USA), 1:600 (rabbit polyclonal antibody; Santa Cruz Biotechnology) and 1:800 (rabbit polyclonal antibody; Abcam, Cambridge, UK). The empirical procedure was performed based on the manufacturers’ instructions.

#### 4.2.2. Assessment Standard

We considered a result to be positive if there were buffy granules in the cell membrane and cytoplasm. According to the chromatosis intensity, no pigmentation, light yellow, buffy and brown were scored as 0, 1, 2 and 3, respectively. Chromatosis cells were observed with the whole section. Chromatosis cells that accounted for <5% were 0; 5%–25%, 1; 26%–50%, 2; 51%–75%, 3; and >75%, 4. Multiply these 2 numbers: 0 to 2 is considered (−); 3 to 4 (+); 5 to 8 (++); and 9 to 12 (+++). The 86 cases of OCCC were divided into the CD44 high-expression group (++/+++) and the CD44 low-expression group (−/+) and similarly for CD47 and c-met. Two observers analyzed the sections to control for error.

### 4.3. Statistical Analysis

Differences in proportions were evaluated using the χ^2^ test. The χ^2^ test or Fisher’s exact test was used to analyze the relationship between CD44, CD47 and c-met expression and clinicopathological variables, whichever was appropriate. Survival curves were generated using the Kaplan–Meier method and compared by the log-rank test. Cox’s proportional hazard regression model was used for multivariate survival analysis of the prognostic factors. All statistical analyses were performed using SPSS version 17.0. (SPSS Inc., Chicago, IL, USA) A two-tailed p-value test was used in all analyses, and *p* < 0.05 was considered statistically significant.

## 5. Conclusions

Expression of CD44, CD47 and c-met was upregulated in OCCC and pairwise correlation. CD44, CD47 and c-met may have synergistic effects on the development of OCCC and are prognostic factors for ovarian cancer.

## Supplementary Materials

Supplementary materials can be found at http://www.mdpi.com/1422-0067/16/02/3391/s1.
